# Ocular Ultrasound as a Key to Diagnosing Uveitis-Masked Syndromes: Tips and Tricks

**DOI:** 10.3390/clinpract15050084

**Published:** 2025-04-23

**Authors:** Valeria Albano, Rosanna Dammacco, Ilaria Lolli, Claudia Ventricelli, Enrico Settimo, Angelo Miggiano, Maria Grazia Pignataro, Paolo Ferreri, Francesco Boscia, Silvana Guerriero, Giovanni Alessio

**Affiliations:** 1 Department of Basic Medical Sciences, Neurosciences and Sensory Organs, Eye Clinic, University Hospital Polyclinic of Bari, Piazza Giulio Cesare 11, 70124 Bari, Italy; 2 Department of Basic Medical Sciences, Neurosciences and Sensory Organs, Eye Clinic, University of Bari, Piazza Giulio Cesare 11, 70124 Bari, Italy

**Keywords:** masquerade syndrome, uveitis, ultrasonography, intraocular lymphoma, multimodal imaging, vitritis, Toxoplasma, scleritis, uveal melanoma, metastasis

## Abstract

**Background and Objectives**: Uveitis-masked syndromes or masquerade syndromes (UMSs) are a group of ocular conditions with several systemic underlying causes, malignant or nonmalignant, that mimic the inflammatory status of the uvea. They are often difficult to detect and diagnose with traditional techniques, such as ophthalmic exams. Ocular B (bidimensional)-ultrasound (OBU) is a non-invasive, repeatable, rapid ultrasound method effective in indirect signs that lead back to systemic diseases. It is comparable in effectiveness with other imaging tools. The cause of UMSs can often be serious, and therefore early diagnosis and prompt treatment are critical. This study aimed to identify the sonographic signs of these forms, which can help physicians discover the cause underlying UMS. **Materials and Methods**: This was a consecutive, retrospective, nonrandomized study. This study was conducted at the University Hospital Polyclinic of Bari, Italy, from January 2022 to December 2024. A total of 186 patients were included, from 10 to 85 years old. They all underwent B-scan ultrasonography (Quantel Medical ABSolu Ocular Ultrasound). **Results**: All patients reported blurred vision, which could be accompanied by visual reduction (<20/40, Snellen charts), photophobia, floaters, flashes, proptosis, and redness. In all cases, we noted peculiar ultrasonographic signs, which allowed us to discriminate the underlying systemic diagnosis, such as vitreous corpuscles, choroid thickening, and primitive or metastatic solid tumors. Finally, we identified different diseases, such as primary intraocular lymphoma (PIOL), other lymphoproliferative conditions, orbital plasmacytoma, uveal melanoma, metastasis, endogenous endophthalmitis, retinal detachment, central serous retinopathy, metallic foreign bodies, ocular amyloidosis, and drug-induced UMSs. The sensitivity and specificity of ocular ultrasound compared to multimodal ocular imaging in UMSs were as follows: for primary intraocular lymphoma (PIOL) and other lymphoproliferative conditions, 0.98 (95% CI, 0.80–1) and 0.68 (90% CI, 0.40–0.92), respectively; for orbital plasmacytoma, 0.64 (92% CI, 0.52–0.86) and 0.66 (93% CI, 0.48–0.89), respectively; uveal melanoma, 1.00 (98% CI, 0.88–1.00) and 0.98 (95% CI, 0.86–0.98), respectively; metastasis, 0.75 (95% CI, 0.53–0.85) and 0.85 (95% CI, 0.48–0.98), respectively; endogenous endophthalmitis, 1.00 (95% CI, 0.50–1.00) and 0.83 (95% CI, 0.48–0.98), respectively; retinal detachment, both were 1.00 (95% CI, 0.87–1.00 and 0.84–0.97, respectively); central serous retinopathy, 0.60 (80% CI, 0.41–0.88) and 0.85 (95% CI, 0.52–0.98), respectively; metallic foreign bodies, 1.00 (95% CI, 0.78–1.00) and 0.99 (95% CI, 0.99–1.00), respectively; ocular amyloidosis, 0.77 (82% CI, 0.52–0.90) and 0.83 (80% CI, 0.49–0.88), respectively; and drug-induced UMSs, 0.64 (95% CI, 0.49–0.88) and 0.85 (95% CI, 0.52–0.98), respectively. **Conclusions**: Diagnosing UMS accurately can be quite challenging, and many of its different types frequently go undetected. This complexity in identification often leads to underdiagnosis, meaning it is essential to improve awareness and understanding of the condition in order to achieve better recognition and treatment. Early detection of these forms is imperative. The use of OBU can help diagnose indirect signs of these forms early and treat them promptly. It compares well with other diagnostic imaging techniques, such as MRI, but this does not mean that it replaces them; it can offer added value in multimodal imaging.

## 1. Introduction

We speak of masked forms when uveitis, i.e., the inflammation of the uvea, presents with atypical symptoms or when the inflammation is masked by other eye diseases.

In ophthalmology, the term “masquerade syndrome” or uveitis masquerade syndrome (UMS) describes malignant and non-malignant conditions that mimic intraocular inflammatory processes.

It was first used in 1967 by FH Theodore to describe conjunctival carcinoma presenting as chronic conjunctivitis [1].

The UMSs are a group of systemic and ocular disorders characterized by the appearance of inflammatory signs, more specifically of intraocular infiltrating cells, and chronic symptoms that are unresponsive to conventional treatment. Conventionally, UMSs are divided into two distinct categories: malignant masquerade syndromes and non-malignant syndromes. The former encompasses both systemic and intraocular neoplasms. At the same time, the latter comprises a wide range of benign non-inflammatory conditions such as an intraocular foreign body, retinitis pigmentosa, ocular amyloidosis, ocular ischemic syndrome, and retinal detachment. The UMSs are considered in differential diagnosis from infectious uveitis, such as toxoplasmosis, sarcoidosis, tuberculosis, syphilis, and birdshot chorioretinitis.

The prevalence of UMS is low in the general population, compounded by misdiagnosis and underdiagnosis. Two retrospective epidemiology studies analyzed UMS prevalence.

A study conducted by Rothova et al. at a tertiary center revealed that the prevalence of UMS among patients presenting with inflammatory syndromes was 6%. Among the sample, 1.8% were confirmed as malignant UMS, while the remainder were benign conditions mimicking inflammatory diseases [2].

The Grange study documented a similar prevalence of UMS, reporting a rate of 2.5%. This finding is supported by the National Eye Institute’s data, which indicate that 21 out of 853 patients diagnosed with uveitis between 2004 and 2012 also met the diagnostic criteria for a neoplastic masquerade syndrome [3].

Patients presenting with uveitis manifestations tend to be older in comparison with those presenting with uveitis for the first time. Furthermore, uveitis is most frequently observed among females. However, this prevalence in gender is not revealed in cases of UMS [3].

Although UMS is rare, it is imperative to be aware of the clinical conditions and presentations that mimic uveitis and to arrive at an early diagnosis of the masquerade pathology, to preserve visual acuity.

In cases of neoplastic masqueraders, early diagnosis has been proven to be a critical factor in survival outcomes, as it can potentially prevent the progression of cancer and save lives [3].

### 1.1. Systemic Diseases’ Related Masquerade Syndrome

Primary intraocular lymphoma (PIOL) continues to be the most common neoplasm that can mimic nonspecific uveitis.

Historically known as reticulum cell sarcoma, PIOL is a rare form of non-Hodgkin’s high-grade B-cell lymphoma, although rare T-cell variants have also been described. PIOL has a wide range of presentations.

Most commonly, patients present with painless vision blurring. At ophthalmoscopic evaluation, cellular inflammation into the vitreous humor can be found, resembling an intermediate uveitis. This can be associated with subretinal creamy yellow infiltrations or deep subretinal infiltrations, while it is very rare to find involvement of the anterior segment.

Early diagnosis is critical to improve survival chances, as up to 80% of PIOL cases have CNS involvement [4].

Much rarer is ocular secondary involvement in patients with systemic lymphoma, which is usually represented by uveal lesions.

Much more frequent, although always rare in the general population, is ocular involvement in leukemia, which usually presents as direct retinal lymphoblastic infiltration [5].

The orbit can also be the site, albeit rare, of plasma neoplasms, such as orbital plasmacytoma. Orbital plasmacytoma is a particular plasma cell-proliferative condition of monoclonal immunoglobulin chains from differentiated B cells. It is present with non-specific signs as orbital cellulitis, exophthalmos, diplopia, and chemosis [6].

Ocular metastasis and primary uveal melanoma only rarely masquerade as uveitis. The presence of uveitis in the context of a primary or secondary tumors, manifesting in either the anterior chamber or the vitreous as vitritis, has been documented. Pigmented cells from intraocular melanomas may also disperse and be wrongly identified as inflammatory cells [7,8].

Additionally, cases of scleritis and choroidal effusion have been reported. They can present the first time as UMS [9].

Retinoblastoma, the most frequent malignant intraocular tumor in childhood, has been documented to present as mimicking different conditions. Orbital cellulitis-like presentations, as well as tumor seeds disseminating in the anterior chamber and mimicking iridocyclitis, have been reported in the literature.

Diffuse infiltrating retinoblastoma is also known to closely resemble anterior uveitis, as it is prone to presenting with anterior chamber seeding and pseudohypopyon, along with a mass with calcifications in the posterior segment, with vitritis [10].

Among non-malignant conditions that present with symptoms like those of uveitis, endophthalmitis is of particular significance. It is imperative to differentiate between these two conditions in a timely manner, as endophthalmitis necessitates prompt treatment to mitigate the risk of visual impairment.

While acute endophthalmitis presents with specific clinical signs, chronic endophthalmitis, post-operative or post-traumatic, has a more insidious onset with the involvement of both the anterior chamber and the vitreous [11].

Retinal detachment, if long-standing, can present accompanied by synechiae, anterior uveitis, and vitritis [12].

Central serous chorioretinopathy mimic uveitis but is often mistaken for posterior uveitis, with the consequence of mistakenly starting a corticosteroid therapy [13].

The presence of metallic foreign bodies in the eye, if not promptly resolved, can result in mechanical and chemical injuries presenting at a later stage as unilateral steroid-resistant uveitis accompanied by siderotic coloration of the iris and vitreous. Pigment dispersion syndrome is characterized by pigmented deposits on the corneal endothelium and atrophic areas on the iris, which can lead to misdiagnosing it as herpetic uveitis [14].

Ocular amyloidosis can be misdiagnosed as vitreous as it usually appears with bilateral cobwebs, sheet-like veils, or strings of pearl-like white opacities [15].

Especially in elderly patients, a dense vitritis from Toxoplasma gondii infection may mask a primary lymphoma of the central nervous system. Therefore, in such cases, it is mandatory not only to test for Toxoplasma IgG antibodies but also to perform cytofluorimetry and brain magnetic resonance imaging (MRI) to rule out lymphoma [16].

Similarly, sarcoidosis, tuberculosis, syphilis, Behçet’s disease, and birdshot chorioretinitis can also present with vitritis, and it is important to distinguish between these forms by means of laboratory tests, chest X-rays, and computer tomography (CT) scans [17].

Some drugs can cause vitritis simulating UMS. Among these, in the literature, there are reports of prostaglandin analogs, metipranolol, corticosteroids, cholinomimetics, brimonidine, and certain antibiotics [18]. Moreover, some intraocular drugs cause uveitis, such as brolucizumab, with it appearing a few weeks after intraocular injection [19].

It is also known that certain antineoplastic drugs can mimic uveitis, such as vemurafenib (anti-BRAF), dabrafenib (anti-BRAF), nivolumab (anti-PD-1), ipilimumab (anti-CTLA-4), osimertinib (anti-EGFR), trametinib (anti-MEK), and rituximab (anti-CD20) [20,21]. Therefore, greater care should be taken to observe cancer patients diagnosed with lymphoma, metastatic cutaneous melanoma, lung adenocarcinoma, metastatic thyroid carcinoma, and pleomorphic xanthoastrocytoma [20,21].

### 1.2. Symptoms

UMSs usually mimic intermediate uveitis, so they manifest with the same symptoms, which are floaters and blurred vision. In addition, they can present at onset with other highly variable symptoms, which make initial diagnosis very difficult. These are signs that are common to uveitis, such as redness, pain, eyelid edema, and cellulitis. Diagnostic tests can be indispensable in these cases to identify the underlying disease.

### 1.3. Multimodal Imaging and Other Tests

Multimodal imaging can be useful in the diagnosis of UMSs. The choice of multimodal imaging and other diagnostic tests to be used depends on the patient’s clinical presentation and the physician’s diagnostic suspicion. Optical coherence tomography (OCT) of the retina and optic nerve is particularly useful in identifying early changes in masked syndromes, such as retinal thickening or the presence of fluid under the retina. Optical coherence tomography angiography (OCT-A), which is a technique that combines OCT with angiography, can be useful in identifying vascular abnormalities that may be associated with UMS, such as vasculitis. Fluorangiography (FA) is used to visualize fluid leaks or other abnormalities in the blood vessels that may be associated with UMSs. Fundus autofluorescence (FAF) can be useful to identify changes in the retina that may be associated with UMS such as macular degeneration and atrophy. Anterior segment imaging (AS-OCT) can be useful to identify alterations that may be associated with masked syndromes, such as iris inflammation.

In the various forms in which UMS can manifest, multimodal imaging tools are complementary techniques, and ultrasound is useful because the lesion is more posterior (sclera and orbit) and there is greater media opacity.

However, multimodal imaging in the UMSs has an unavoidable requirement that there should be no opacities that could hinder the examination. Therefore, in forms of UMS, the single or combined use of most multimodal imaging modes is prevented by situations of opacities of intraocular structures. This causes the results of examination by multimodal imaging to be partial or undefinable in most cases.

These examinations are also affected by opacities, so the examination is often not entirely effective at reaching a timely diagnosis.

Beyond these, laboratory tests are useful to identify especially infectious forms of UMSs. In addition, neuroimaging modes, such as MRI and CT scans, are often considered to identify the underlying cause of UMS. A CT scan of the globe and the orbit is used to detect metallic foreign bodies.

In this regard, a very important examination for PIOL forms is MRI, allowing the analysis of cerebral spinal fluid, as one-third of patients have lymphoma cells and vitreous specimens showing a high IL-10-to-IL-6 ratio in cytokine analysis.

### 1.4. Role of B-Scan Ocular Ultrasound

Ocular B-scan ultrasound (OBU) is a handy diagnostic tool, especially when the uses of other imaging techniques are limited. Frequently, this imaging technique is used whenever the dioptric media are not clear, such as in cases of corneal opacities, Tyndall phenomena in the anterior chamber, hemorrhages into the anterior chamber or vitreous cavity, cataracts, pupillary seclusion with synechiae, vitreous haze, and satellite retinal detachment. In most of these cases, other multimodal imaging techniques based on the passing of light do not display intraocular structures. Opacification of the dioptric media may compromise the accuracy of a fundoscopic examination because, in the presence of opacities, posterior ocular structures are not adequately highlighted. Unlike other examinations, ultrasound can overcome media opacities [22].

OBU studies vitreous, choroidal, and retinal abnormalities, as well as solid bulbar and orbital tumors, allowing a more accurate and earlier diagnosis [23,24,25].

### 1.5. Benefits and Limits of B-Scan Ocular Ultrasound

This diagnostic method has some benefits as well as some disadvantages. The advantages include being a non-invasive diagnostic technique, safe, with no radiation, and repeatable several times without a risk to the patient [26].

Among the disadvantages are a lower resolution than other techniques, such as optical coherence tomography (OCT), for example, and operator dependence (the quality of the examination depends partly on the experience of the operator) [26].

## 2. Methods

This was a consecutive, retrospective, nonrandomized study. This study was performed in accordance with the current version of the Declaration of Helsinki (52nd WMA General Assembly, Edinburgh, Scotland, October 2000). A total of 186 consecutive patients referred to our Eye Clinic of the University Hospital Polyclinic of Bari, Italy, between January 2022 and December 2024 were included. Initially, they all complained of blurred vision accompanied by visual defects, photophobia, floaters, redness, ocular pain, photophobia, and tearing at hospitalization. All patients underwent B-scan ultrasonography (Quantel Medical ABSolu^®^ Ocular Ultrasound, Lumibird Medical, Cournon-d’Auvergne, France) with a 12 MHz B probe.

The inclusion criteria were all patients with ages between 10 and 85 years and visual acuity impairment of less than 20/40 (Snellen charts). Patients who had previously undergone cataract surgery, vitrectomy, or trabeculectomy for glaucoma were also included. The exclusion criteria were terminal glaucoma, diabetic retinopathy, and rhegmatogenous retinal detachment in the earlier stages. Written informed consent was obtained from all patients and their parents. Institutional Review Board/Ethics Committee approval was obtained.

We considered the sensitivity and specificity of OBU for different MSU diagnoses to assess their accuracy compared with MRI. Sensitivity is the ability to correctly identify true positives, reducing the risk of false negatives. Specificity represents the ability to correctly identify true negatives, reducing the risk of false positives. Both are critical in assessing the reliability of a diagnostic test.

## 3. Results

The main characteristics of the population of this study are summarized in Supplementary Table S1, which includes the age distribution, gender percentages, and number of each type of UMS, true positives, true negatives, false positives, and false negatives.

During our clinical investigations, when subjecting all patients to OBU, we noticed that patients with a certain diagnosis had overlapping ultrasound pictures, which led us to focus on certain findings. We realized that the ultrasound element common to most was the vitreous corpuscles and assessed that, in some diagnoses, these could be associated with the other characteristic ultrasound patterns listed below.

The sensitivity and specificity of ocular ultrasound compared to multimodal ocular imaging in UMSs were as follows:

For primary intraocular lymphoma (PIOL) and other lymphoproliferative conditions, 0.98 (95% CI, 0.80–1) and 0.68 (90% CI, 0.40–0.92), respectively; for orbital plasmacytoma, 0.64 (92% CI, 0.52–0.86) and 0.66 (93% CI, 0.48–0.89), respectively; uveal melanoma, 1.00 (98% CI, 0.88–1.00) and 0.98 (95% CI, 0.86–0.98), respectively; metastasis, 0.75 (95% CI, 0.53–0.85) and 0.85 (95% CI, 0.48–0.98), respectively; endogenous endophthalmitis, 1.00 (95% CI, 0.50–1.00) and 0.83 (95% CI, 0.48–0.98), respectively; retinal detachment, both were 1.00 (95% CI, 0.87–1.00 and 0.84–0.97, respectively); central serous retinopathy, 0.60 (80% CI, 0.41–0.88) and 0.85 (95% CI, 0.52–0.98), respectively; metallic foreign bodies, 1.00 (95% CI, 0.78–1.00) and 0.99 (95% CI, 0.99–1.00), respectively; ocular amyloidosis, 0.77 (82% CI, 0.52–0.90) and 0.83 (80% CI, 0.49–0.88), respectively; and drug-induced UMSs, 0.64 (95% CI, 0.49–0.88) and 0.85 (95% CI, 0.52–0.98), respectively.

False positive and false negative numbers for all UMS types are also included in Supplementary Table S1.

### B-Scan Sonography Patterns

Vitreous patterns: The vitreous appeared with abundant corpuscular movements (vitreous corpuscles), and the posterior vitreous seemed organized into blocks or clouds, with or without vitreous detachment. Vitreous corpuscles could be classified as follows: 1. mild (finely dispersed forward of the posterior vitreous), 2. moderate (coarsely organized on the posterior vitreous, with or without highly reflective membranes), 3. severe (abundant haze located in the entire vitreous cavity forward of the posterior vitreous with or without highly reflective membranes).

In the UMSs, vitreous corpuscles could comprise inflammatory cells, purulent inflammatory cell, tumor cells, or vitreous metastasis. The intensity of the vitreous corpuscles or their mobility alone could not allow us to pinpoint the UMS (inflammatory, infectious, neoplastic, metastatic). Nevertheless, we noted that more frequently infectious forms (sarcoidosis, tuberculosis, candidiasis) could result in a moderate or severe form, even in association with hyperreflective membranes. Unlike the other infectious forms, however, ocular infection with toxoplasma gondii caused an ultrasound picture of severe vitreous corpuscles but not associated with hyperreflective membranes, and the focus of infection was often not revealed by B-scan ultrasound.

Posterior exudation: This was aligned with the retinal profile with vitreous adherences or organized in the vitreous cavity in single or multiple foci. Among these, we found some forms of intraocular lymphoma or systemic lymphoma, and some bacterial or fungi infections, always associated with vitreous corpuscles.

Serous retinal detachment: This was frequently primitive or secondary to a solid tumor. It was normally associated with neoplasms, either primitive or metastatic.

Choroid thickening: This was typical of sarcoidosis.

Choroidal detachment: This was usually associated with inflammatory or infectious conditions.

Corpuscles of the vitreous and/or thickening of the choroid or extrinsic muscles: This formed the common sonographic picture of lymphoma and other lymphoproliferative conditions.

Solid tumor associated with vitreous and/or retina and/or choroid and/or extrinsic muscles and/or other orbital structures: This was typical of neoplasms, such as intraocular or orbital lymphoma, melanomas (associated with vitreous corpuscles, especially in the necrotizing form), metastasis, retinoblastoma, leukemia, and other lymphoproliferative conditions. Some solid tumors may have growth toward the vitreous. These types of tumors arose in differential diagnosis with foreign bodies, which instead may be suspended in the vitreous or lying on the posterior profile, characterized by sonographic posterior shadowing.

Moreover, this technique led us to detect some complications, such as identifying retinal detachments, neovascularization, and other structural changes caused by inflammation.

It is mandatory to review the history of the patient, their age, the anatomical location, and their clinical symptoms in order to form a suspicion of UMS.

When we had a suspected underlying cause, we also performed serological, radiographic, MRI, CT, tissue biopsy, and vitreous sampling laboratory tests to confirm the diagnosis. For example, in a suspected PIOL vitrectomy sample, with cytofluorimetry, cytokine level tests, brain MRI, etc. Elderly patients with a history of lung or breast cancer or hematological disorders must be closely watched. If a malignant nature was suspected based on the ultrasound findings, endophthalmitis was diagnosed by microbiological assay.

**Figure 1:**
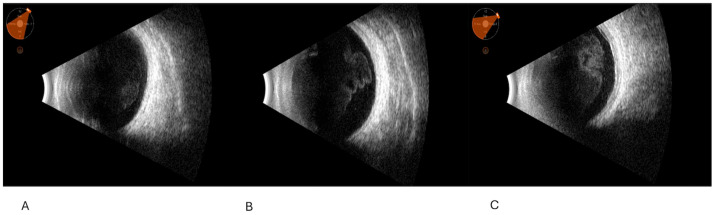
Ultrasound findings in mild, moderate, and severe vitritis. (**A**) Slight vitreous corpuscles collected in “clouds” with minimal increase in chorioretinal thickness (syphilis); (**B**) moderate vitreous corpuscles with a high mobility, organized in posterior clusters. The main aspect of the vitreous body is its high cellularity, which is accompanied by abnormalities of the posterior profile (tuberculosis); (**C**) abundant vitreous corpuscles with structured free hyperreflective membranes in the vitreous, associated with multiple posterior profile abnormalities, including posterior membranes with subretinal fluid, and thickening in the macular region (birdshot chorioretinopathy with choroidal neovascularization, CNV).

**Figure 2:**
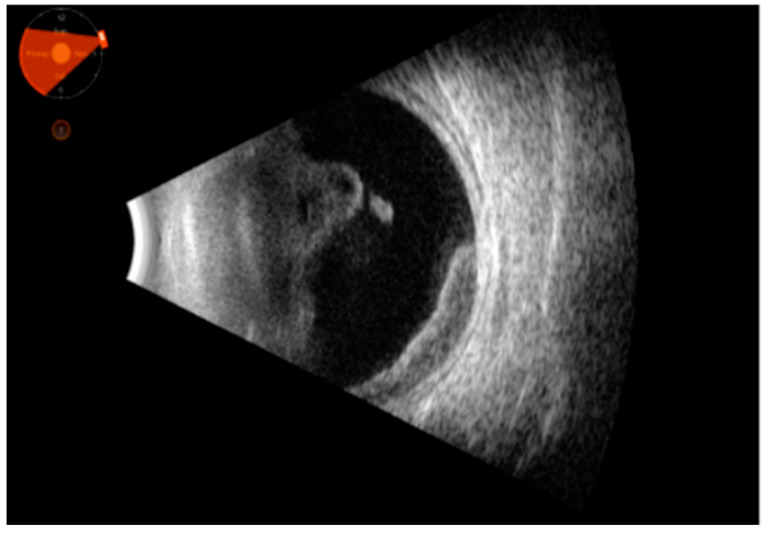
Ultrasound picture of abundant vitreous corpuscles organized into clusters in the anterior vitreous, with high cellularity and mobility. Moreover, the picture shows a highly cellular structure with medium–high reflectivity in the temporal sector, at times in continuity with the vitreous (intraocular lymphoma).

**Figure 3:**
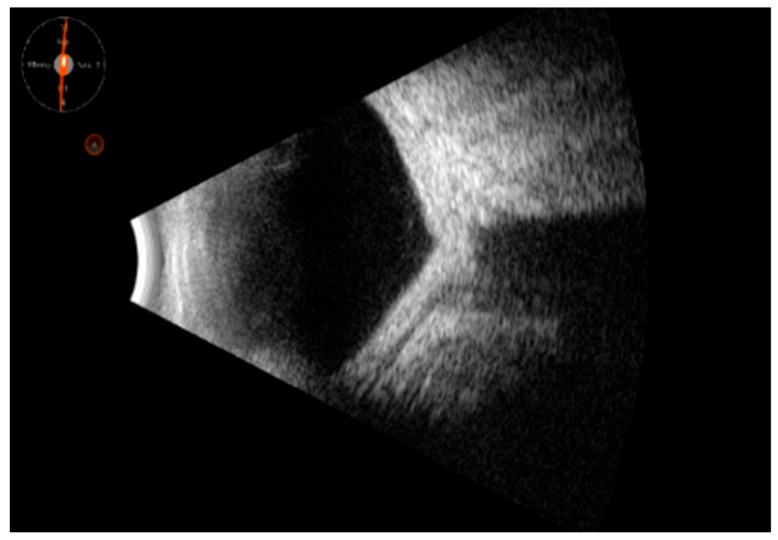
Complete annular hematological cellular infiltration of the tenon capsule and sclera, with a “sleeve-like” appearance, also involving the posterior pole (plasmacytoma); none of vitreous abnormalities are shown.

Representative images are given in Figure 1, Figure 2 and Figure 3.

## 4. Discussion and Conclusions

UMSs encompass a wide spectrum of diseases that can mimic uveitis, presenting clinicians with considerable challenges in diagnosis and management. UMS should be considered as a differential diagnosis in all patients presenting with undifferentiated or treatment-resistant ocular inflammation.

Despite the extensive use of laboratory and imaging techniques, the accurate diagnosis of these conditions can be delayed, resulting in delayed initiation of appropriate treatment for these patients. For retinal specialists, multimodal imaging techniques such as OCT, OCTA, FA, and FAF are not useful in cases with opaque media.

Often, this is due to ocular conditions that do not allow eye structures to be examined, such as dense opacities (cataract, corneal leucoma, vitreous haze, etc.). The use of B-scan ultrasonography can help physicians distinguish among the different forms of UMSs, allowing an earlier diagnosis compared other imaging tools, such as MRI [27].

The sensitivity and specificity of OBU in detecting MRI-confirmed optic nerve swelling were 100% (CI 95% 54.1–100) and 58.3% (CI 95% 27.7–84.8), respectively, according to the literature, based on the accuracy of the optic nerve diameter [28].

OBU is not intended to replace other diagnostic examinations in UMSs. Still, it can allow a suitable initial discrimination to recognize different types of UMSs thanks to its high sensitivity and specificity compared with other multimodal images and the advantage of overcoming the opacity limit of dioptric media, which other multimodal imaging media—OCT, OCTA, FA, FAF—cannot overcome.

As a non-invasive tool requiring little or no cooperation from the patient, it has proved very useful for all ages, especially in children, where cooperation may be absent or poor [29].

The use of B-scan ultrasonography in UMSs proves very useful in the first instance in emergency settings and for hospitalized patients, even when they have complicated and difficult systemic conditions, including poor mobility [30]. Accordingly, the use of OBU in the emergency room has greatly increased, as reported for American insurance claims in 2018 [31].

Moreover, it has proven very useful in UMSs caused by drug treatments, and in monitoring various therapies during the follow-ups [32,33].

OBU is a powerful diagnostic tool in the evaluation of UMS. Its practical and timely use allows for a prompt and accurate diagnosis, which is crucial in preventing further complications and initiating appropriate treatment. This non-invasive imaging technique provides a detailed visualization of the eye’s internal structures, enabling the identification of subtle abnormalities that may be missed during a standard clinical examination. However, while OBU is invaluable, it should not be used in isolation. To establish a definitive clinical diagnosis, it is often necessary to complement OBU findings with other diagnostic modalities, such as laboratory tests to assess inflammatory markers, radiological examinations to rule out other potential causes, and in some cases, sampling of the aqueous humor and vitreous for further analysis. This comprehensive approach ensures that all possible contributing factors are considered, leading to a more precise diagnosis and a tailored treatment plan for the patient.

While B-scan ultrasonography has proven valuable in evaluating UMS, we encountered limitations in its practical application. A significant challenge lies in the substantial operator expertise required for accurate interpretation. Differentiating between various underlying causes, such as tumors, retinal detachments, or infectious processes, demands extensive experience in sonographic pattern recognition. Furthermore, precisely localizing abnormalities within the complex ocular structures affected by masquerade syndrome can be difficult even for seasoned sonographers. Subtle variations in echogenicity and anatomical relationships may be missed by less experienced operators, potentially leading to misdiagnosis or delayed treatment. This reliance on highly specialized skills underscores the need for rigorous training programs and continuous quality assurance measures to maximize the diagnostic yield of B-scan ultrasonography in these challenging cases. Future advancements in automated image analysis and standardized scanning protocols may help mitigate these limitations and improve the accessibility of this valuable diagnostic tool.

## Data Availability

The original contributions presented in this study are included in the article/Supplementary Materials. Further inquiries can be directed to the corresponding author.

## Supplementary Materials

The following supporting information can be downloaded at https://www.mdpi.com/article/10.3390/clinpract15050084/s1, Table S1: Characteristics of the population of study.
