# Supplementary material for: Ocular Ultrasound as a Key to Diagnosing Uveitis-Masked Syndromes: Tips and Tricks
Source: Clin Pract. 2025 Apr 23;15(5):84. doi: 10.3390/clinpract15050084 (PMC12110100; doi:10.3390/clinpract15050084)
Supplement: Supplementary file 1 [file clinpract-15-00084-s001.zip › clinpract-3540360-supplementary.pdf]

Table S1. Characteristics of the population of study.

	All (N=186)	true positive	true negative	false positive	false negative
**Age distribution, years (range)**	10-85				
**Gender (M/F)**	70/116				
**Primary Intraocular Lymphoma (PIOL)**	15	15	0	7	3
**Systemic Lymphoma**	6	6	0	3	1
**Orbital Plasmacytoma**	1	1	0	1	1
**Uveal Melanoma**	16	16	0	4	2
**Metastasis**	12	12	0	4	0
**Endogenous Endophthalmitis**	31	31	0	5	0
**Retinal Detachment**	6	6	0	2	0
**Central Serous Retinopathy**	5	5	0	1	4
**Metallic Foreign Bodies**	14	14	0	4	0
**Ocular Amiloidosis**	2	2	0	3	1
**Retinoblastoma**	2	2	0	2	1
**Drug-induced UMS**	15	15	0	11	1
